# Evoking transitions from states of motor readiness to motor action in a phase-coupled oscillator model of motor cortex

**DOI:** 10.1186/1471-2202-12-S1-P19

**Published:** 2011-07-18

**Authors:** Stewart Heitmann, Pulin Gong, Michael Breakspear

**Affiliations:** 1 School of Psychiatry, The University of New South Wales, Randwick, NSW 2031, Australia; 2 Black Dog Institute, Randwick, NSW 2031, Australia; 3 School of Physics, The University of Sydney, Camperdown, NSW 2006, Australia; 4 Faculty of Medicine, The University of Sydney, Camperdown, NSW 2006, Australia; 5 Queensland Institute of Medical Research, Herston, QLD 4029, Australia; 6 Royal Brisbane and Women's Hospital, Herston, QLD 4029, Australia

## Background

Rapid changes in behavior require rapid changes in brain states, yet the brain must also remain stable in the face of noise and uncertainty. The problem is exemplified by motor readiness in which execution of a planned motor behavior is voluntarily delayed until the onset of a 'go' stimulus. Achieving such rapid changes between otherwise stable brain states remains a fundamental problem in computational neuroscience. Single-state attractor networks provide stability but require global changes to the attractor landscape to alter network state and such transitions are invariably slow. Multi-state attractor networks promise rapid transitions between co-existing stable states by direct perturbation of the state variables however the nature of the perturbation is critical. Here we explore the efficacy of a global perturbation for invoking state transitions in a bistable model of motor cortex based on phase-coupled neural oscillators [1].

## Methods

Motor cortex was modeled as a two-dimensional sheet (128x128) of phase-coupled neural oscillators that were locally coupled using a center-surround connection topology. This model exhibits bistability when the strength of the inhibitory surround is within a specific range. In such cases the oscillators spontaneously self-organize into either spatially synchronous patterns or spatiotemporal traveling wave patterns depending upon initial conditions. These patterns are reminiscent of those observed in primate motor cortex during voluntary [2] and self-paced movements [3] where the timing of the evoked waves are dependent on both the onset of movement and the phase of the local-field potential [2,3].

## Results

We show that a global perturbation that is sinusoidally coupled to the phase of the mean-field can evoke a rapid transition from the synchronous state to the wave state. Our results demonstrate that an appropriately timed global perturbation can induce rapid transitions between distinct stable firing patterns and we posit that motor cortex may use a similar mechanism to transition rapidly from motor readiness to action.

## References

[B1] Ermentrout B Neural networks as spatio-temporal pattern-forming systems Rep Prog Phys 1998 61 4 353 430 10.1088/0034-4885/61/4/002

[B2] Rubino D Robbins KA Hatsopolous NG Propagating waves mediate information transfer in the cortex Nat Neurosci 2006 9 12 1549 1557 10.1038/nn1802 17115042

[B3] Reimer J Hatsopolous NG Periodicity and evoked responses in motor cortex J Neurosci 2010 30 34 11506 11515 20739573 10.1523/JNEUROSCI.5947-09.2010 PMC2952881

